# Adaptability and sustainability of machine learning approaches to traffic signal control

**DOI:** 10.1038/s41598-022-21125-3

**Published:** 2022-10-06

**Authors:** Marcin Korecki

**Affiliations:** Computational Social Science, ETH Zurich, Stampfenbachstrasse 48, 8092 Zurich, Switzerland

**Keywords:** Computational science, Computer science

## Abstract

This study investigates how adaptable Machine Learning Traffic Signal control methods are to topological variability. We ask how well can these methods generalize to non-Manhattan-like networks with non-uniform distances between intersections. A Machine Learning method that is highly reliable in various topologies is proposed and compared with state-of-the-art alternatives. Lastly, we analyze the sustainability of different traffic signal control methods based on computational efforts required to achieve convergence and perform training and testing. We show that our method achieves an approximately seven-fold improvement in terms of CO$_2$ emitted in training over the second-best method.

## Introduction

Due to the continuous increase of the global population and rising migration from rural areas into urban environments, there emerges a necessity for the evolution of the concept of the city. As cities become bigger, denser, and more populated the solutions to problems such as traffic control that were previously efficient become ineffective^1^. At the same time, new challenges caused by this paradigm shift exacerbate the limitations of the old model of the city. An answer to these issues is the new paradigm of the smart city, where the traditional infrastructure is combined with information and communications technology and coordinated via emerging digital technologies^2^.

One of the key issues of the modern city is its environmental impact on the immediate surroundings as well as its citizens. Urban pollution’s detrimental health effects^3^ as well as its negative impact on perceived happiness^4^ have been well-documented. As such, one of the main goals of the new concept of a city—a smart city is to allow for creation of technologies that can be integrated into the city infrastructure for greater efficiency and sustainability in order to create cities that are not only smart but also sustainable^5^.

A key contributor to the lack of urban sustainability is the common transportation model relying on motorized vehicles. The frequent congestion of traffic causes increased emissions, loss of time, and sound pollution. Moreover, noise and air pollution caused by vehicles are considered key indicators of pedestrian perception of urban spaces^6^. Therefore, improving the current transportation paradigm is of crucial importance for the development of sustainable smart cities^7^.

Some promising approaches addressing the negative effects of traffic are the introduction of electric vehicles and alternative modes of transportation^8^. However, traffic signal control remains the most widespread solution to limiting congestion and increasing the efficiency and sustainability of traffic. A recent trend in the sustainable smart city paradigm is to address traffic congestion with Machine Learning and Internet of Things technologies^9^.

Cities and even more so smart cities are highly dynamic environments, thus they require adaptable approaches. For instance, in order to make the street network more environmentally friendly or to avoid heat island effects^10^ the road network might need to be adjusted (for example to introduce more greenery^11^). Similarly, a recent trend aims at rethinking the streets themselves to, among other things, reduce the number of cars allowed on them^12^. Machine Learning shows great promise as a traffic signal control approach, yet the training of advanced models is computationally expensive which often translates into environmental costs^13^. Moreover, most proposed Machine Learning methods are trained on particular city topology and traffic flow and might not readily adapt to temporal and spatial changes. A model requiring retraining when the topology changes or the training of a separate model for different parts of the city would require significant resources and would not be sustainable. Thus a good traffic control model needs to be able to adapt to changes in the road network at least to some extent.

**Figure 1:**
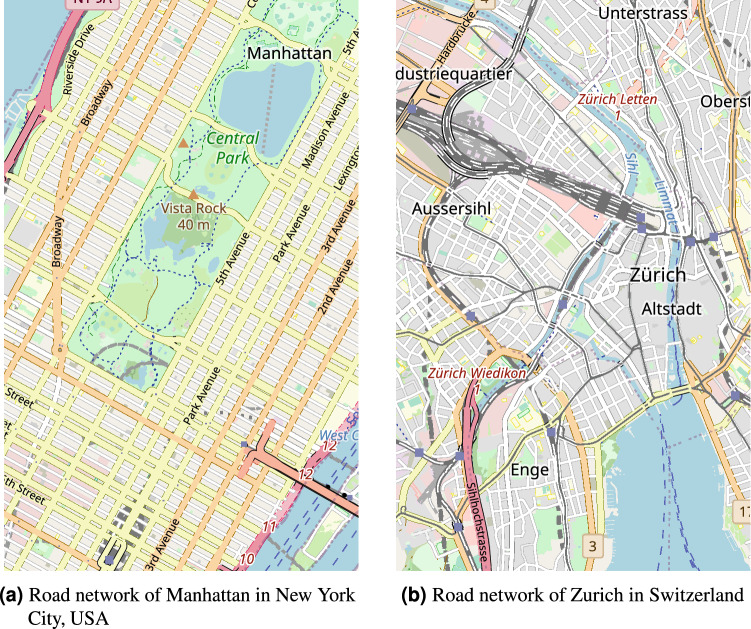
A comparison of the road networks of two large cities, note the difference between the grid like structure of Manhattan and the more chaotic network of Zurich^16^.

We note that most Machine Learning approaches to traffic signal control focus on idealized Manhattan-like grids which are not representative of the cities in, for example Europe (see Fig. 1)^14,15^. It is important to investigate how well these models generalize to less idealized conditions of more heterogeneous networks.

In this paper we introduce a novel, Machine Learning solution to the problem of traffic signal control, that does not require frequent and costly retraining, and compare it with state-of-the-art alternatives. We show that a clever design of the state space of a Reinforcement Learning (RL) algorithm, along with pretraining the algorithm on a simple scenario yields an adaptable and highly efficient model. In the interest of sustainability, we not only consider the efficiency of the compared methodologies but also their environmental impact. Furthermore, we study how well different traffic signal control methods adapt to changes in the road network topology and compare their results on non-Manhattan-like road networks. We conclude that the proposed method is able to outperform alternative classical and RL approaches in terms of average travel time achieved due to its length-invariant state design (able to account for heterogeneous road lengths) and analytic exploration. Moreover, the method boasts a superior environmental efficiency. do to employing a pretraining procedure.

## Background

In this section, we formulate the description of the traffic signal control problem as well as define the Reinforcement Learning approach to it.

### Glossary of terms

**Figure 2:**
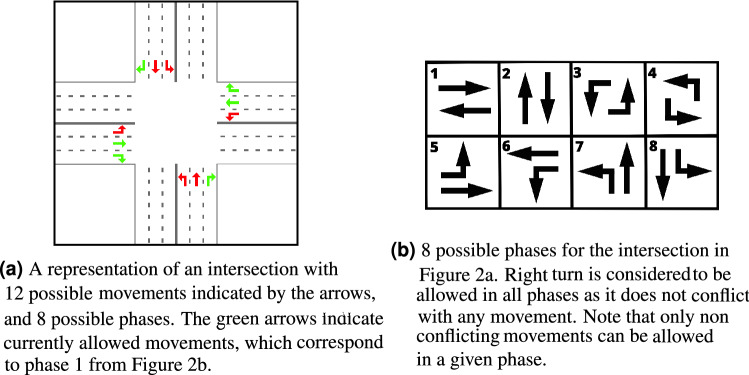
A representation of an intersection with 12 possible movements indicated by the arrows, and 8 possible phases. The green arrows indicate currently allowed movements, which correspond to phase 1 from Fig. 2b. 8 possible phases for the intersection in Fig. 2a. Right turn is considered to be allowed in all phases as it does not conflict with any movement. Note that only non conflicting movements can be allowed in a given phase.

- **Movement**: Represents traffic flow moving through an intersection from an incoming lane to an outgoing lane. The movement from lane *a* to *b* is denoted as (*a*, *b*). The intersection in Fig. 2a consists of 12 movements, each indicated with an arrow.
- **Phase**: Represents the set of all movements that are allowed at a given time (given green light). In Fig. 2a, the phase allows for 6 movements indicated in green and corresponding to phase 1 in Fig. 2b.
- **Movement Pressure**: Represents the imbalance between the number of cars of all incoming lanes of the movement and the number of cars on all outgoing lanes of the movement, adjusted for the maximum number of cars possible on the lane^17^, according to Eq. (1).
  1 $$\begin{aligned} w(l,o) = \dfrac{x(l)}{x_\mathrm{max}(l)} - \dfrac{x(o)}{x_\mathrm{max}(o)} \, . \end{aligned}$$
  where *w*(*l*, *o*) is the pressure of movement (*l*, *o*), *x*(*l*) represents the number of cars on incoming lane *l*, *x*(*o*) the number of cars on the outgoing lane *o* and $x_\mathrm{max}(l)$ and $x_\mathrm{max}(o)$ corresponds to the maximum number of cars possible on lanes *l* and *o*.
- **Intersection pressure**: represents the imbalance of cars on outgoing and incoming lanes across all the possible movements of the intersection, following Eq. (2).
  2 $$\begin{aligned} P_i = \left| \sum _{(l, o) \in i} w(l,o)\right| \, , \end{aligned}$$
  Here $P_i$ is the pressure of the intersection *i* and the rest follows the notation from Eq. (1).

### Reinforcement learning for traffic signal control

Arguably, the most common Machine Learning approach to traffic signal control is Reinforcement Learning^18^. Each intersection of a given road network is associated with a Reinforcement Learning agent which observes the state of the intersection. The data representing the observed state at the particular decision step is fed to the agent, which then selects an action, that is the phase to be set. The goal of the agent is to select the actions so that some reward function is maximized. Depending on the design of the agent, the state and reward definitions might differ significantly leading to different results. A detailed review of Reinforcement Learning approaches to the traffic control problem can be found in^19^.

## Related literature

In this section, we present some of the relevant literature on the topic of traffic signal control.

### Conventional

The traditional method of controlling traffic lights in cities is to employ cyclic traffic plans, rotating over the phases^20^. In more advanced versions of this approach, the cycles are enacted with offsets and the green times given to each phase may vary^20^. An improvement over the cyclical plans is the SCOOT system (split, cycle and offset optimization technique), which is a data-driven method that is able to adapt to changing and unusual conditions^21^. Further improvements around adaptive methods included prioritization of public transport and privileged vehicles^22^. At the beginning of the 21st century the conventional methods have begun to be replaced by more intelligent and real-time measurement based solutions. An example of such a method would the hierarchical control architecture—RHODES that decomposed the control problem into several sub-problems and included a module for prediction of traffic flow^23^.

Due to the high variability of urban traffic conventional approaches are usually ineffective in avoiding the creation of traffic jams and have been consistently outperformed by the methods related below.

### Self-organizing

The goal of the self-organizing approaches to traffic signal control is to achieve emergent optimization by allowing individual agents to control their behavior in a decentralized manner^24^. The control mechanism is often designed so that negative interactions between agents are avoided and cooperation is maximized.

One self-organizing approach relies on an analytic model of traffic derived from queuing theory^25^ and base decision making algorithm on two rules: an optimization and stabilization rule. The optimization rule relies on a short-term anticipation of future arrivals and calculating the minimum green time needed to clear a queue. The stabilization rule makes sure the algorithm avoids situations where a large queue overflows to the previous intersection. A control scheme based on this method has been successfully deployed in Dresden, Germany, and Lucerne, Switzerland^26,27^.

### Reinforcement learning

Many recent traffic signal control algorithms rely on Deep Reinforcement Learning^18^. They differ in their state and reward description as well as the particular type of learning algorithm used. The main learning algorithms in Reinforcement Learning are value-function learning and policy gradient and they have both been applied to traffic signal control^28^. An example of the value-function learning would be any algorithm that relies on a DQN (Deep *Q*-network), an example of a policy gradient method would be DDPG (Deep Deterministic Policy Gradient). The key difference between the two is that DQN outputs discrete actions, while DDPG is able to output continuous actions. Furthermore, DQN requires training of one neural network while DDPG, being an actor-critic algorithm requires at least two networks to be trained (the actor network and the critic network and often two additional target networks).

The design of the reward function has a crucial effect on the efficiency of the algorithm, which has been studied in some depth^29^. One of the approaches to the reward design is to use the concept of pressure (Eq. 2)^14^. Similarly, the design of the state space is of great importance^29^, depending on its design the algorithm might be limited to work only for intersections with a particular number of movements^30^. An example of state description is to use the queue length, the number of vehicles, the waiting times and an image representation of the intersection^31^. Some research has also investigated how the state description can be structured, including prior knowledge to achieve better learning outcomes^30^. An important distinction is weather the agents operate independently, or share some information in a centralized manner. In^32^, the authors compare different decentralized algorithms such as traditional *Q*-Learning and advantage actor critic in the context of traffic signal control. Some publications have highlighted the benefits of agents sharing information with neighbors^33^ or sharing the neural network parameters in training and deployment^14^. One recently proposed method, GuidedLight, aimed to combine the benefits of Reinforcement Learning with analytic approach by injecting analytic knowledge into the exploration procedure of the Reinforcement Learning agent^15^.

An overview of the recent state-of-the-art Reinforcement Learning methods can be found in^34,35^. These Reinforcement Learning approaches show great promise and convincing results in simulated homogeneous, grid-like environments. It is not clear, however, how well these models can adapt to changes in the topology of the road-network or how well they perform in non-Manhattan like settings.

### Meta-reinforcement learning

A limitation of classical Deep Reinforcement Learning is that the learned models, while achieving good results in the scenarios they were trained on, tend to lack the ability to generalize to different scenarios. In a sense the neural networks at the core of Deep RL can overfit to the data that is provided to them in training. When they later, in deployment, encounter data that they have not been exposed to in training they might recommend actions that are not optimal. The main goal of task generalization is to design learning models that are able to generalize to unseen environments^36^. Since in RL it has been a standard practice to train and test the models on the same environment it is not clear what is the models’ (including the ones in traffic signal control) ability to generalize^37^. Training and testing in different environments is one of the ways of addressing the issue and so we employ it in our experiments.

Another method that has been designed to address the task generalization is Meta-Learning. It deals with methods that can generalize well to an array of scenarios^38^. It is worth noting that Deep *Q*-Learning (or value-function learning) has been shown to possess some Meta-Learning qualities if given access to a context variable that represents the past trajectory^39^. Approaches that show increased adaptability to changing traffic flows have already been proposed. One way is to use a gradient-based model agnostic meta-learning^40^ for periodically alternating global and individual adaptation and leveraging the experience gotten from learning different scenarios^41^. In a similar manner, better generalization can be achieved by leveraging model agnostic meta-learning and flow clustering^42^. A recent study has implemented a meta learning model-based framework to address the need to improve data efficiency and limit the number of interactions with the environment that are needed to learn an efficient model^43^. Another approach to meta-learning has focused on incorporating both short and long term information in the learning process through the use of decentralized advantage actor-critic approach^44^. On the other hand, a solution based on a meta learning spatial-temporal graph attention network along with a long short-term memory unit has been proposed to deal with neighbor information processing^45^. Most of the meta-learning methods proposed in recent years can be characterized by a rather high complexity of the proposed models. The methods differ in the exact area they focus on (neighbor information, data efficiency etc.) as well as in the way they could be implemented in real world (centralized, decentralized). However, little attention has been given to adaptation to topological variability of the scenarios, which we aim to address in this paper.

## Methods

In this section, we will describe in detail the implementation of the proposed agent, that controls the traffic signal at an intersection, and the experimental setup used to showcase and compare its results. As the basis for our proposed agent, we use the GuidedLight agent, which has been shown to perform favorably when compared to other Machine Learning methods as well as analytic self-organizing methods^15^. We have decided to focus on Deep *Q*-learning methods as they have been shown to achieve comparable results to other methods such as DDPG^46,47^. Furthermore, DQN requires training of fewer deep neural networks as compared to DDPG (which needs an actor network, a critic network and often two additional target networks), since we are interested in decreasing the environmental impact of training the models it makes sense to focus on models which require less computational time to be trained.

We will introduce the features of the proposed agent’s state, exploration and learning algorithm design. We will then introduce the novel pretraining procedure and summarize the proposed experimental design.

### Length invariant state design

The usual state design of the Reinforcement Learning agent for traffic signal control includes the observations available to a single intersection. This most often consists of the number of cars in the incoming lanes, as well as the number of cars on the outgoing lanes along with the current phase of the intersection. Since the aim is to create an agent which can adapt well to a variety of different road network topologies, we wish to make the state as invariant to topological heterogeneity as possible.

Our aim is to make the agent perform well not only on intersections where the length of incoming and outgoing lanes is the same but also on ones where these lengths vary. In real-life conditions, especially in European cities (Fig. 1b), it is common that the distances and so lengths of roads between intersections are not uniform. Because of that, we propose to express the number of cars on in/outgoing lanes, not as an absolute number but rather the proportion of the lane that is occupied. A 50-m long lane with five 5-m cars on it would be covered in 50%, on the other hand, a 25-m long lane with the same cars would be 100% covered. Even though both lanes have the same number of cars waiting on them it makes more sense to prioritize the one that is full to avoid an overflow. Our agent needs to distinguish between such cases so that it can work well in situations where the lanes have non-uniform lengths. Hence, we introduce the length invariant state design where the occupancy of the lane is expressed in terms of coverage and not simply the absolute number of cars on it.

### Analytic exploration

Like all Reinforcement Learning approaches, GuidedLight agent needs to explore its environment before the model becomes sufficiently effective. Exploration is more likely to happen in the early stages of training and the chance that it occurs decreases with time. Instead of exploring randomly (choosing random actions instead of actions suggested by the model), GuidedLight uses analytic exploration ($\alpha$-exploration). Rather than taking random actions at times, it takes actions that would be selected in a given state by an analytic algorithm^25^. This approach not only speeds up convergence but also leads to better results.

### Deep *Q*-learning

GuidedLight agent implements the Deep *Q*-Learning approach. The learning process is based on a *Q*-function mapping state and action pairs to the expected reward. This function is learned using function approximation capabilities of a deep neural network. The mapping is based on the expected future reward as in Eq. (3).

3 $$\begin{aligned} Q^{new}(S_t, A_t) = Q(S_t, A_t) + \ell \cdot (R_t + \gamma \cdot \max _{A}Q(S_{t+1}, A) - Q(S_t, A_t)) \end{aligned}$$

Here $Q^{new}$ is the *Q*-value after an update for the state-action pair ($S_t, A_t$ at time *t*), *Q* is the previous *Q*-value for the state-action pair, $\ell$ represents the learning rate and $\gamma$ weights the effect of short-term vs. long-term gains. $R_t$ is the reward at time *t* and $\max _{A}Q(S_{t+1}, A)$ is the estimate of the highest obtainable *Q* value if one starts from state $S_{t+1}$ and selects optimal actions. In Deep *Q*-Learning, the *Q*-function is approximated using a Deep *Q* Network (DQN). The GuidedLight Agent implements the Double Deep *Q*-Network (DDQN) approach for greater learning stability^48^.

### GuidedLight agent

The agent controls the phase of a single intersection in the traffic network. GuidedLight design is as follows.

- **State**: consists of the percentage coverage of each of three equal segments of the incoming lanes, the coverage of the outgoing lanes and the current phase (one-hot-encoded). The separation of incoming lanes into segments was shown to improve results^14^.
- **Actions**: consist of all possible phases at the given intersection.
- **Reward**: based on the concept of pressure of the intersection as in Eq. (2) and following Eq. (4).
  4 $$\begin{aligned} R_i = -P_i \end{aligned}$$

where *R* is the reward of intersection *i*. By maximizing the negative of its pressure the agent balances the number of cars between its incoming and outgoing lanes which leads to maximization of throughput and minimization of average travel time^17^. An individual GuidedLight agent is deployed at each intersection, however, the agents share the same DQN and store and replay the memory from a shared buffer. This data sharing speeds up the convergence and leads to better results^30^. Thus in a scenario with 16 intersections there would be 16 agents deployed. These agents, however, would share the same DQN and would add their experience to the same memory replay that would then be available to all other agents. The further details of the implementation of the GuidedLight agent are found in the following sections.

### Pretraining

**Figure 3:**
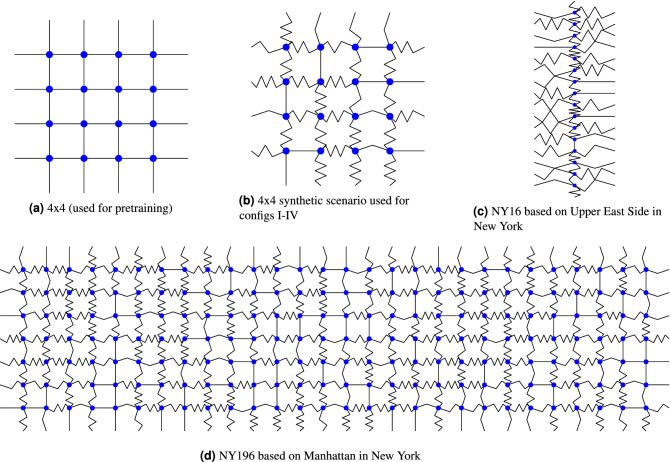
The four road networks used in the experiments, blue dots indicate intersections, black lines indicate roads. Note the varied lengths of the roads in (**b**–**d**).

To study GuidedLight’s generalization ability we train the model on a synthetic, uniform, Manhattan-like road network. The network is shown in Fig. 3a and the flow is the same as for scenario I (Table 1). The trained model is then tested on the non-uniform and more complex scenarios with different topologies and different traffic flows. The model only learns on the simple network and no further learning occurs in subsequent tests. This way we can assess GuidedLight’s resilience to changing conditions.

### Experiments

To test the GuidedLight agent’s efficiency and performance we conduct a series of experiments. First, we compare the average travel time, which is considered to be a good indicator of overall performance^14,31,41,42^, achieved by GuidedLight on six different simulated scenarios with the results of state-of-the-art alternatives, which we have chosen based on their good performance. We follow with an ablation study where we compare GuidedLight’s performance on our most complex scenario—NY196—against versions of GuidedLight that do not use analytic exploration and/or the improved state design. Lastly, we conduct a comparison of the training times of each of the compared methods with their results on the NY196 scenario as well as convergence analysis for the learning algorithms. The non-pretrained, learning methods are trained on each scenario separately and the results of the best model tested on the corresponding scenario is reported.

**Table 1:** Traffic flows and road lengths assumed in the simulation scenarios.

Configuration	Arrival rate (vehicles/s)	Demand pattern (arrival rate variance)	Average road length (m)
I	0.388	0.3	$447.23 \pm {115.82}$
II	0.388	0.6	$447.23 \pm {115.82}$
III	0.416	0.3	$447.23 \pm {115.82}$
IV	0.416	0.6	$447.23 \pm {115.82}$
NY16	1.89	0.00008	$397.42 \pm {133.00}$
NY196	0.803	0.0011	$425.79 \pm {108.72}$

The details of the road networks used for the experiments and their flows can be found in Table 1. A visualization of the road networks’ topologies is presented in Fig. 3. The regular topologies of the networks have been disrupted by elongating the roads at random leading to a situation, where a single intersection has non-uniform lanes’ lengths.

The NY16 scenario follows real-world traffic flow data from Upper East Side^14^ but with a modified network topology. Similarly, NY196 follows real-world data from Manhattan^33^ with a modified topology of the network. We are especially interesting to test how the compared methods scale with the increase in the number of intersection. Hence the NY196 scenario is of most interest to us as it represents a scale at which these methods could be deployed in real life.

The learning agents in this study take decisions every 10 s. When changing phases, 2-s all-red signal (allowing only right turns) is emitted. All scenarios are run for 1800 simulation steps corresponding to 30 min of real-time. Cityflow simulator^49^ is used for all experiments. The learning agents are trained for 150 learning epochs, where one epoch is a full run of the 1800 simulation steps. The DQN used for GuidedLight and PressLight has two layers of 128 and 64 nodes, learning rate of 0.0005 with Adam optimizer is used for training. The training uses mini-batches of 64 samples, the memory is limited to 10000 data points and $\gamma$ is set to 0.8. The parameters have been selected through grid search. The Simple DQN and FRAP agents use the parameters from^50^.

### Compared methods

- **FixedTime**: static method, rotating through all possible phases one after another, included for comparison^20^.
- **Random**: an agent choosing actions at random, a safety baseline below which no learning algorithm should fall.
- **Analytic**: an advanced analytic method based on short term traffic anticipation and self-organization, implements an optimization and stabilization rule^51^.
- **SimpleDQN**: a simple Reinforcement Learning method^32^ using a Deep *Q*-Network. Each agent uses a different network and the parameters are not shared.
- **FRAP**: a state-of-the-art Reinforcement Learning method^30^, that uses state design invariant to symmetrical cases, thus reportedly being able to generalize to more diverse situations.
- **PressLight**: a state-of-the-art Reinforcement Learning method^14^. It uses a regular DQN (not the double DQN), random exploration., and absolute number of cars on incoming and outgoing lanes in the state.
- **Pretrained PressLight**: a PressLight model trained on the synthetic $4\times 4$ scenario.
- **GuidedLight**: a Reinforcement Learning method described above, relying on analytic exploration and length invariant state description. Key differences between PressLight and GuidedLight are the exploration procedure, state design and the use of DDQN instead a DQN.
- **Pretrained GuidedLight**: a GuidedLight model trained on the synthetic $4\times 4$ scenario.

The code used for all experiments and agents (except Simple DQN and FRAP which are available https://github.com/mKafouros/PlanLighthere) is available at https://github.com/mbkorecki/rl_trafficthis repository.

## Results

In this section, we present the results of the experiments outlined in the previous section.

### Overall performance

In Table 2 we present the results of the tested methods on the 6 experimental scenarios in terms of the average travel time achieved by the methods. The table shows that the best results for most settings are achieved by the Pretrained GuidedLight model. Only in the NY16 scenario does the result of the Pretrained GuidedLight share first place with PressLight. This might be caused by the fact that for the NY16 scenario each intersection has at most two neighbors. Due to that, the problem of overflow, which GuidedLight addresses with it length invariant state design, is less pronounced as the negative interactions between neighbors are less likely. For all settings except for NY16 GuidedLight is the second best. It is also worth noting that the analytic algorithm’s performance is exceptionally high and in most scenarios within 1–2% of the best result. Furthermore, we note that in 5 out of 6 scenarios the Pretrained PressLight achieves worse results than PressLight, only in NY196 is the Pretrained version better, but by a very small margin. The situation is different with GuidedLight, where the pretrained version is significantly superior to the non-pretrained version in all scenarios. Moreover, it is clear that the NY196 scenario, being the most complex one, shows the biggest differences between the algorithms. It is also apparent that two of the ML methods—SimpleDQN and FRAP—perform surprisingly bad on the tested scenarios. In fact the performance of both these methods is worse than the performance of the Random and Fixed Time methods. Thus, we do not include these methods in the training, test time and convergence experiments.

**Table 2:** Average travel time of different traffic signal control methods for 4 configurations of $4\times 4$ synthetic scenario and two real-world based scenarios.

Model	Avg. travel time (s)					
	I	II	III	IV	NY16	NY196
Fixed time	547.40	480.45	547.05	491.20	511.12	774.37
Random	502.17	466.43	509.30	485.51	424.41	765.45
Analytic	285.90	287.31	291.07	305.48	261.82	669.00
SimpleDQN	619.09	607.85	661.13	636.20	609.5	–
	(10.70)	(11.13)	(13.63)	(8.52)	(9.25)	
FRAP	560.82	520.75	538.33	526.14	534.84	–
	(22.61)	(15.10)	(37.22)	(22.36)	(16.48)	
PressLight	288.65	278.36	296.96	297.42	243.84	706.36
	(1.87)	(2.08)	(2.66)	(2.15)	(4.31)	(3.93)
Pretrained PressLight	289.77	280.43	300.17	314.26	287.98	697.71
GuidedLight	274.36	270.03	289.25	293.46	255.95	660.58
	(1.78)	(3.06)	(1.98)	(2.96)	(2.48)	(5.77)
Pretrained GuidedLight	**271.44**	**265.55**	**277.07**	**285.84**	246.71	**647.45**

The values in brackets are standard deviations of the last 10 training epochs. Pretrained methods do not include standard deviations as they are trained and tested on different scenarios. Best results are presented in bold, second best are underscored. The NY196 scenario has not been run with FRAP and Simple DQN as the implementation of these agents is prohibitively slow and energy consuming for large scenarios^50^.

### Ablation study

In Table 3, we report the results of the ablation study. We can see that the removal of the length invariant state description (GuidedLight-% state) leads to significantly worse results. The same is true of the removal of $\alpha$-exploration. Finally, the removal of both of these features leads to the worst results. Interestingly, it seems that the removal of the length invariant state description also increases the standard deviation of the last 10 training epochs which can be interpreted as the training process being less stable. It is worth noting that GuidedLight without % state and $\alpha$-exploration is still better than PressLight, showcasing the benefits of using Double Deep *Q*-Network.

**Table 3:** Results of the ablation study on the NY196 scenario.

Model	Avg. travel time (s)	
GuidedLight -$\alpha$-exploration -% state	688.8	(9.33)
GuidedLight -$\alpha$-exploration	682.97	(5.23)
GuidedLight -% state	684.66	(9.34)
GuidedLight	**660.58**	(5.77)

Bold Indicates result with the lowest average travel time.

-% state indicates a model using absolute number of vehicles instead of road length adjusted coverage, -$\alpha$-exploration indicates a model using random exploration. The values in brackets indicate the standard deviations of the last 10 training epochs.

### Training/test times and convergence

**Figure 4:**
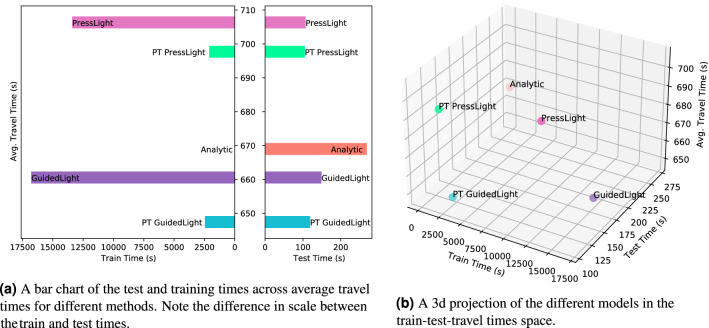
Training and test times vs. the avg. travel time on the NY196 scenario. PT stands for pretrained.

In Fig. 4, we report the test and train times for the compared models as well as their results in terms of average travel time. All models were run on a cluster using AMD EPYC 7742 CPU and requiring at least 8 cores, 2 GeForceRTX2080Ti GPUs and 2048 MB of RAM. As can be seen, the models have varied train time with GuidedLight requiring most training time and analytic requiring no training at all. On the other hand, at test time the analytic algorithm is most time-consuming.

It is important to note that the main source of the computational strain for the GuidedLight and the analytic algorithm is the calculation of the rates of arrival and departure. Due to the design of the simulation, computing these values requires some costly operations. In deployment, such values can be easily detected using inductive loops^52^. Thus the computational costs in the deployment of GuidedLight would be much closer, if not the same, as PressLight’s. Similarly, the analytic test time cost would become lower in deployment.

**Figure 5:**
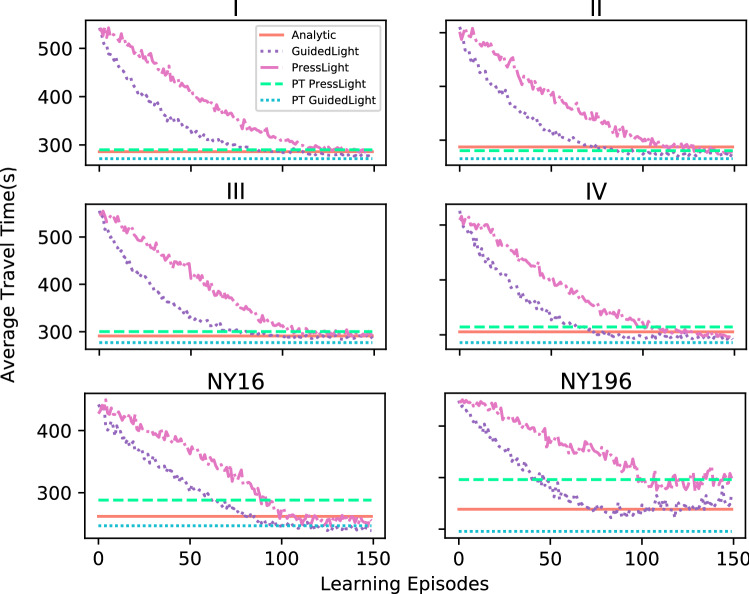
Average travel time achieved through learning epochs for PressLight and GuidedLight along with the results achieved by the non-trained methods: analytic and two pretrained (PT) models.

In Fig. 5, we report the convergence for training learning algorithms along with the results of the pretrained or non-learning approaches. We can see that for the synthetic scenarios the final results are similar for all compared methods. The largest differences occur in the NY196 scenario. It is interesting to note that the performance of the analytic algorithm in the NY196 is very high and that the GuidedLight trained on this scenario takes a around 80 epochs to converge to the analytic results and even the best model barely surpasses the analytic results. Lastly, it can be seen that GuidedLight converges faster and towards better results than PressLight.

## Discussion and conclusion

In this paper, we proposed a Machine Learning agent for traffic signal control that achieves, in the majority of the scenarios studied, better results than both state-of-the-art Machine Learning and analytic alternatives. Moreover, we have shown that our agent shows high levels of topological adaptability. Namely, a model pretrained on a simple setting with uniform distances between intersections achieved results surpassing all compared alternatives when run on more complex scenarios with non-uniform distances between intersections. We showed that PressLight does not appear to adapt well to changes in topology, since the pretrained version of PressLight performed worse than PressLight. At the same time pretrained GuidedLight was shown to perform better than the non-pretrained version showcasing its topological adaptability. The success of the pretraining procedure in case of GuidedLight can be explained by its length invariant state design, which allows it to generalize its experience to different topological conditions. On the other hand PressLight is not able to perform such generalization as it will experience completely different states in different topological conditions. Our ablation study further confirmed the power of proposed improvements, both the length invariant state as well as the $\alpha$-exploration approach. We also showcased the faster convergence of the GuidedLight agent over PressLight, which is due to the effects of $\alpha$-exploration.

Moreover, we compared the training and test times of the studied methods. These values can be understood as indicators of the environmental impact of the methods as computational time translates into energy expenditure^13^. Using a Machine Learning CO$_2$ calculator^53^ we can perform some high level approximation of the actual amount of carbon emitted during training. We assume the world average value of carbon efficiency of 475 g/kWh from 2019^54^. Under these assumptions the training of GuidedLight on the NY196 scenario would emit 0.56 kg of CO$_2$ which is equivalent to burning 0.28 kg of coal or driving 2.26 km with an average car. On the other hand using the pretrained GuidedLight would yield only 0.08 kg of CO$_2$ corresponding to burning 0.04 kg of coal or driving 0.32 km with an average car. That is 7 times less CO$_2$ when using the pretrained model, a significant improvement. Furthermore the analytic solution would emit no CO$_2$ altogether as it does not require training. It is worth noting that depending on the country where the computational power is located and the potential offsets employed these values may vary significantly. These calculations stress the importance of limiting the number of times the models need to be retrained and furthermore showcase how one can decrease the training costs by employing methods such as pretraining on a simpler scenario.

We noted the high training and test costs of GuidedLight but also noticed that the Pretrained GuidedLight’s testing and training times are very low. Thus the proposed pretraining procedure not only allows for great adaptability but also increases the environmental friendliness of the method. Why train the model on a highly complex setting if it can adapt to it better after being trained on a simple, less demanding scenario. It is also important to note that the analytic method while performing worse than GuidedLight does not need any training. In some cases, it might be more beneficial and sustainable to deploy such a method even if there is a slight drop in efficiency (in terms of avg. travel time for instance).

We also note the apparent lack of effectiveness of the SimpleDQN and FRAP methods, which, while being consistent with the results in other work^50^ (where both these methods perform worse than Fixed Time in real-world scenarios), is still surprising. This low performance is likely due to the methods focusing on fully individual agents, with no parameter sharing or communications. Such agents require much more training data to learn optimal behavior. These findings reflect the need for strong baselines that can be used to judge the performance of ML methods. In a similar vein it reflects the need of varied testing scenarios. For example FRAP has been shown to perform better than some baselines in some simple scenarios^30^, but in more complex, realistic scenarios it appears to falter, as shown in this work.

With this work we hope to contribute to the trend of sustainability-conscious Machine Learning, which has recently seen growing interest. While we see the great potential of the Machine Learning for the particular problem of traffic signal control, exemplified by our results, we also believe it important to quantify and weight the environmental costs of such methods. Future directions for study include further investigating the pretraining procedure and identifying what properties of the smaller network used for pretraining lead to high adaptability of the model as well as considering models that are adaptable to disruptions in both topology as well as traffic flows.

In summary, we have presented and compared the topological adaptability and sustainability of some Machine Learning and analytic methods. We have shown the value of pretraining and intelligent state design as well as the benefits of injecting analytic knowledge into the Machine Learning models via exploration. Lastly, we were able to propose an adaptive and sustainable Machine Learning method—the Pretrained GuidedLight which boasts superior performance to the alternatives.

## Data Availability

The datasets generated and analyzed during this study are available from the corresponding author on reasonable request. The code used to run the experiments is available https://github.com/mbkorecki/rl_traffichere.
